# Pre-analytic assessment of dried blood and dried plasma spots: integration in mass spectrometry–based metabolomics and lipidomics workflow

**DOI:** 10.1007/s00216-025-05760-z

**Published:** 2025-02-05

**Authors:** Eleonora Bossi, Simone Serrao, Pierluigi Reveglia, Antonietta Ferrara, Marta Nobile, Elena Limo, Gaetano Corso, Giuseppe Paglia

**Affiliations:** 1 Department of Medicine and Surgery, Proteomics and Metabolomics Unit, University of Milano-Bicocca, 20854 Vedano al Lambro, Italy; 2 Department of Clinical and Experimental Medicine, University of Foggia, 71122 Foggia, Italy

**Keywords:** Microsampling, Dried blood spots, Metabolomics, Lipidomics, Method optimization

## Abstract

Microsampling, especially dried blood spots (DBS), emerged in recent years as a viable alternative to conventional blood collection since it is rapid, simple, minimally invasive, and has user-friendly characteristics. Moreover, DBS are able to avoid analyte degradation thanks to their great stability. Due to their versatility, clinical applications with DBS have increased, including mass spectrometry–based metabolomics and lipidomics studies. In this work, we evaluated and optimized extraction protocols testing five different extraction solutions to perform metabolomics and lipidomics studies on the same spot considering three commercially available microsampling devices, Capitainer, Whatman, and Telimmune. Parallelly, we also evaluated the short-term stability of the three devices at room temperature for up to 5 days. Our results showed that pure methanol was the best compromise to simultaneously extract from the same spot both the lipidome and polar metabolome. However, we also propose a two-step protocol combining methanol and water extraction that improves polar metabolite extraction and shows improved reproducibility in Capitainer and Whatman. Short-term stability results highlighted that both polar metabolites and lipids were stable for up to 6 days using the Capitainer device, while with Whatman and Telimmune, some significant variations were observed after 3 days for some classes of metabolites/lipids, suggesting the need for cold-chain storage when working with these devices.

## Introduction

The use of microsamples, particularly dried blood spots (DBS), dates back to the 1960s when Guthrie and Susi implemented them in newborn screening for phenylketonuria. Since then, blood microsampling has gained interest, has become widespread, and has been increasingly used in research [1]. Compared to venipuncture, which is considered the gold standard for blood collection, microsampling is minimally invasive and easier, requires a smaller volume of blood (< 150 μL), and is less stressful for the patient. Moreover, it is a point-of-care device that does not require trained or specialized personnel and represents a paradigm shift towards the concept of self and remote sampling [2]. Overall, blood microsampling is considered a viable alternative to conventional blood sampling also because it has minimal transportation and storage requirements (e.g., no strict cold chain required), a reduced biohazard risk, and less potential for contamination [3]. This method of blood collection is generally straightforward and less costly. In addition, DBS samples are expected to preserve analytes over longer periods of time without degradation [4]. Due to its compatibility with various bioanalytical techniques, especially mass spectrometry (MS), clinical applications of DBS have increased, including in the context of omics sciences such as metabolomics, lipidomics, and proteomics. In this landscape, blood microsampling becomes a key factor to overcome challenges and drawbacks associated with traditional venipuncture, thus facilitating remote sampling and longitudinal studies.

Although there are established protocols for DBS in metabolomics [5–7], the implementation in lipidomics is so far limited [8, 9] and specific procedures for preparing and storing DBS are still lacking systematic optimization [10]. As far as we are aware, there is no information regarding the feasibility of doing both metabolomics and lipidomics analyses on the same spot, as well as any attempts or established protocols for this purpose. In this context, it could be useful to develop a protocol for this analysis, since it would limit the cost of blood collection by using the same spot to perform both the analysis. In addition, it would help to save time by reducing the time required for ultra-high-performance liquid chromatography-MS (UHPLC-MS) analysis.

Another advantage of DBS is improved sample stability [11]. This is important considering the applications of blood microsampling, such as sample collection in rural areas, remote sampling, or in general where there is no access to cold-chain storage systems. Although long-term stability at different storage conditions has been widely investigated [12, 13], only few studies have focused on short-term stability [9, 11].

This study aims to evaluate the performance of three commercially available microsampling devices and to optimize the extraction protocol for DBS and dried plasma spots (DPS) to perform two different metabolomics and lipidomics studies on the same spot. The three devices tested were Whatman 903 Protein Saver Cards, Capitainer B, and Telimmune DUO Plasma Separation Cards. Whatman is the first microsampling device to be invented. It is a type of DBS in which whole blood drops are collected on a paper-based substrate. Capitainer B is a quantitative DBS (qDBS) device that enables quantitative blood collection using a microfluidic system with an exact volume of 10 μL. Telimmune DUO enables plasma separation directly from whole blood drops, thanks to a system of adsorption and filtration layers [14]. The first step was the optimization of the extraction for metabolomics and lipidomics, by evaluating different extraction solvents and solutions already used for DBS analysis. The next step was to optimize the protocol to perform both analyses on the same spot. This raised a question regarding the best extraction solution: for the extraction of polar metabolites, a solution with an aqueous and more polar phase is required. For the extraction of lipids, on the other hand, it is usually better to use a less polar solvent without an aqueous phase, such as pure isopropanol or 1-butanol/methanol. For this reason, we decided to optimize the protocol by adding a consecutive extraction step using the pure solvent for lipid extraction and adding a second extraction step with water to achieve better coverage of polar metabolites. The presence of proteins in the samples is a limitation in metabolomics and lipidomics analysis as they might be responsible for interferences and ion suppression. For this reason, we decided to perform BCA assay to quantify protein concentration in DBS samples extracted with different extraction solutions. Furthermore, the short-term stability of DBS and DPS at room temperature was investigated and the metabolome and lipidome coverage was evaluated. This study is particularly important as it not only refines analytical techniques for metabolomics and lipidomics but also demonstrates significant potential for translation into clinical biochemistry applications, enabling advancements in lipidomics and metabolomics analyses in diagnostic and therapeutic contexts.

## Methods

### Materials

Blood microsampling devices: Whatman 903 Protein Saver Card (Cytiva, Global, Little Chalfont, UK), Capitainer B (Capitainer AB, Stockholm, Sweden), Telimmune DUO Plasma Separation Card (Telimmune, West Lafayette, IN, USA). All extraction solutions and UHPLC solvents were LC–MS grade—LiChrosolv® and were purchased from Merck KGaA (Darmstadt, Germany): water, methanol (MeOH), isopropanol (ISO), 1-butanol, acetonitrile (ACN). Medronic acid and ammonium formate were purchased from Sigma-Aldrich/Merck (Darmstadt, Germany).

### Sample collection

Sample collection was performed at the laboratory of Patologia Clinica Universitaria Policlinico Foggia of University of Foggia. Blood was collected with traditional venipuncture from the same subject without any anticoagulant and 50 μL of whole blood was quickly spotted on each microsampling device. After collection, all samples were dried for 2 h and then stored as follows: samples collected for extraction evaluation were stored at − 80 °C, while those for short-term stability evaluation were left at room temperature (RT) for 1 to 5 days, respectively, before storage at − 80 °C. Samples from day 0 were immediately stored at − 80 °C.

### Sample extraction and stability

Five different extraction solvents were tested in triplicate for each device: pure methanol (CH_3_OH 100%), methanol:water 80:20 v/v (CH_3_OH 80%), methanol:water 50:50 v/v (CH_3_OH 50%), pure isopropanol (ISO), and 1-butanol:methanol 50:50 v/v (BuMe). Two spots with a diameter of 3 mm were punched out of the same Whatman sample, while the 6-mm-diameter disks were removed from both the Capitainer B and Telimmune devices using sterile tweezers and all transferred into fresh 1.5-mL Eppendorf SafeLock tubes and incubated on ice for 30 min. After 400 μL of each extraction solvent was added, the samples were stirred for 20 min at 4 °C in a ThermoMixer Compact (Eppendorf, Hamburg, Germany) and then centrifuged for 15 min at 4 °C at 21,000 g. Supernatants were collected and those from Whatman and Capitainer were filtered post-extraction using 3 K cut-off filters (Millipore Amicon (R) Ultra 0.5 mL, Merck KGaA, Darmstadt, Germany). This step is crucial as it eliminates the extracted hemoglobin that could interfere with the detection of the analytes of interest and damage the chromatographic column. The DPS samples were not filtered since the Telimmune device has a filtration layer, and therefore no additional filtration steps are required. Filtration was performed by centrifuging 3 times at 14,000 g at 25 °C for 15 min. The filtered samples were divided into two equal volumes to analyze polar metabolites and lipids separately and freeze-dried for 2 h at RT using the HetoVac VR-I (A. De Mori, Milan, Italy). Finally, they were reconstituted as follows: 80 μL of ACN:H_2_O (50:50 v/v) for polar metabolites, 80 μL of ISO for lipids. Quality control samples (QC samples) were prepared by pooling 10 μL of each sample in a single Eppendorf tube.

One hundred microliters of RIPA buffer was added to each filter, followed by centrifugation (1 min, 100 g, 25 °C) to recover the retained samples. The BCA protein assay (Merck KGaA, Darmstadt, Germany) was performed on retained samples from the Whatman and Capitainer devices. Standard solutions were prepared as a reference (0, 25, 125, 250, 500, 750, 1000, 1500, 2000 μg/mL) to generate a calibration curve. Then the working reagent solution was prepared by mixing reagents A and B in a 50:1 (v/v) ratio and added to both the standard samples and the diluted samples, and then the samples were incubated in a ThermoMixer Compact at 300 rpm for 30 min at 37 °C. The absorbance of each sample was measured at λ = 562 nm using the NanoDrop™One (Thermo Scientific, Waltham, MA, USA).

To evaluate the short-term stability, the sample extraction protocol described before was applied using 400 μL of CH_3_OH 100% to perform both metabolomics and lipidomics analyses on the same spot.

### Consecutive extraction

A total of six samples were processed from each device. The first three samples for each device were extracted with 400 μL of CH_3_OH 100% according to the previously described protocol. For the remaining three samples, a two-step consecutive extraction was performed. First, 400 μL CH_3_OH 100% was added to extract polar metabolites and lipids from each sample. After this first extraction, the extracted volume was divided into two equal volumes for polar metabolite and lipid analyses. Then, 80 μL of H_2_O was added to the spots already extracted with CH_3_OH 100%. After incubation and centrifugation, the recovered supernatant was added to the volume of polar metabolites. This was done to improve the coverage for polar metabolites. The subsequent steps, filtration, freeze-drying, and resuspension were performed as described in the previous paragraph.

### UHPLC-MS analysis

Samples were analyzed using a UHPLC-MS platform comprising an Agilent 1290 II liquid chromatography system coupled to a quadrupole time-of-flight mass spectrometer (Agilent 6546 LC/Q-TOF – Agilent Technologies, Palo Alto, CA, USA). Chromatographic separation for polar metabolites was achieved using an InfinityLab Poroshell 120 HILIC-Z (2.1 × 150 mm, 2.7 μm) column (Agilent Technologies, Palo Alto, CA, USA) equipped with a UHPLC InfinityLab Poroshell 120 HILIC (2.1 × 5 mm, 2.7 μm) guard column. Mobile phase A consisted of 20 mM ammonium acetate and 5 μM medronic acid in water. Mobile phase B was made of pure acetonitrile. Samples were eluted from the column using the solvent gradient: 0 min 90%B, 1 min 90%B, 8 min 78%B, 12 min 60%B, 15 min 10%B, 18 min 10%B, 23 min 90%B, at a flow rate of 0.4 mL/min. Chromatographic separation for lipids was performed using a CSH ACQUITY Premier C18 (2.1 mm × 100 mm, 1.7 µm) column (Waters, Milford, MA, USA). Mobile phase A consisted of 10 mM ammonium acetate in ACN/H_2_O (60/40 v/v) and 0.1% acetic acid. Mobile phase B was ISO/phase A (90/10 v/v). Samples were analyzed at a flow rate of 0.25 mL/min with the following elution gradient: 0 min 99% A, 1 min 99% A, 1.10 min 60% A, 5 min 20% A, 11 min 20% A, 12 min 1% A, 18 min 1% A, 18.10 min 60% A, 20 min 99% A. Samples were analyzed in triplicate in both positive (2 μL injection volume) and negative (5 μL injection volume) ionization mode. For metabolomics, the resolution was set to 40,000 FWHM with a full scan range of 40–1200 m/z, while for lipidomics it was set to 50,000 FWHM and operated in full scan range of m/z 100–1350. QCs were used to monitor the performance of the analysis and were injected every five samples. At the end of the analysis, five injections of QCs were used to collect MS/MS spectra in data-dependent mode (DDA) using an iterative approach.

### Data analysis

Data acquisition (Agilent Technologies, Santa Clara, CA, USA) was used to control the Agilent 1290 II liquid chromatography and the Agilent 6546 LC/Q-TOF mass spectrometer. MassHunter Profinder (Agilent Technologies, Santa Clara, CA, USA) was used to perform feature annotation.

Five consecutive injections of QC samples (obtained by pooling 10 μL from each sample) in DDA mode were used to acquire MS/MS data and to build the in-house library for polar metabolites and lipids based on accurate mass, MS/MS fragments, isotopic pattern, and retention time, and using online databases as HMDB [15] and METLIN [16].

Then samples analyzed in full scan mode were matched based on mass formula, isotope pattern, and retention time against our in-house database and integrated using MassHunter Personal Compound Database and Library (PCDL) Manager Software (Agilent Technologies, Santa Clara, CA, USA). Univariate and multivariate statistical analysis was performed using MetaboAnalyst 6.0 [17] and GraphPad Prism 9.5 (GraphPad Software, Boston, MA, USA, www.graphpad.com), following data normalization as the sum of the signals and data transformation by log_10_ transformation.

### Score calculation

To determine the optimal extraction method among those tested, we developed a scoring system considering the intensity of each feature in each extracted sample. For each feature, the signal intensity of each sample was divided by the maximum value of the intensity of that feature and expressed as a percentage. Then, the score was estimated based on the percentile. Specifically, we assigned value zero to no detected metabolites/lipids, value 1 at the 1st percentile (signal intensity < 25%), value 2 at the 2nd percentile (25% ≤ signal intensity < 50%), value 3 to the 3rd percentile (50% ≤ signal intensity < 75%), and value 4 to the 4th percentile (signal intensity ≥ 75%). Afterwards, the total score was calculated for each extraction solution as the sum of the scores of each feature for each sample. The total score was then expressed as the average of the scores of the three replicates of each sample.

## Results and discussion

### Extraction optimization

**Fig. 1:**
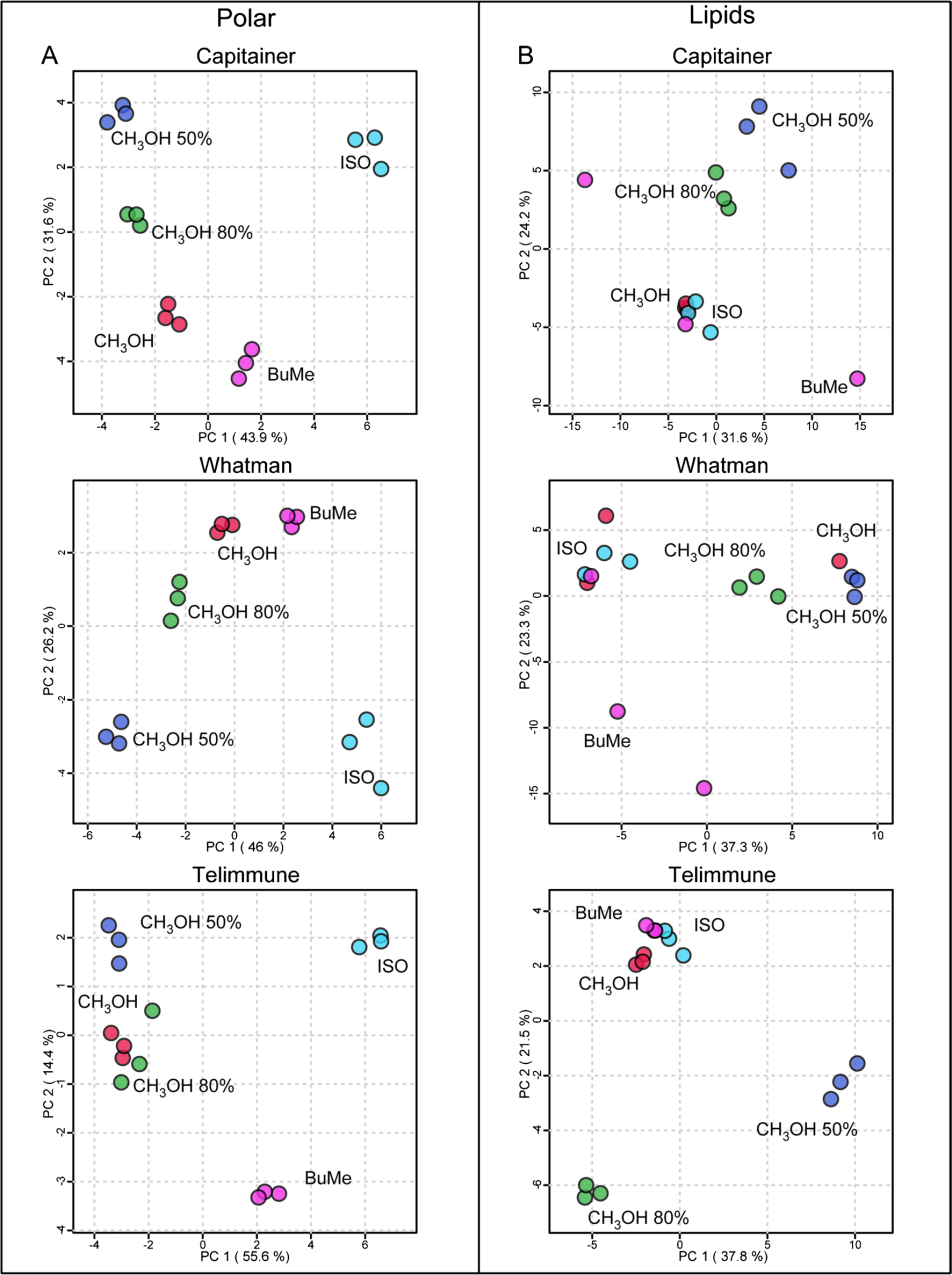
Principal component analysis (PCA) of the five different extraction solutions from polar metabolites (**A**) and lipids (**B**). Samples are assigned a color code considering the different extraction solution (red, CH_3_OH 100%; green, CH_3_OH 80%; blue, CH_3_OH 50%; light blue, ISO; pink, BuMe)

The results of this work provide valuable insights into the efficiency of different extraction methods and their impact on polar metabolome and lipidome from both DBS and DPS. The first step of the method optimization was the choice of extraction solvent. Five different extraction solutions, CH_3_OH 100%, CH_3_OH 80%, CH_3_OH 50%, ISO, and BuMe, were tested to extract polar metabolites and lipids from a single DBS and DPS. As shown by the principal component analysis (PCA) (Fig. 1), the samples were well clustered for polar metabolites in Capitainer, Whatman, and Telimmune, indicating that the five solutions have different extraction yields and a good reproducibility grade. For polar metabolites, the methanol-based solvents clustered close to each other, suggesting similar extraction yields in DPS. The polar scores of both PC1 and PC2 were as percentage 75.5%, 72.2%, and 70% for Capitainer, Whatman, and Telimmune, respectively (Fig. 1). For lipids, however, clustering is less defined, indicating that some extraction solutions have similar extraction performances. The lipid scores of both PC1 and PC2 were as percentage over 55%, 63%, and 59% for Capitainer, Whatman, and Telimmune, respectively (Fig. 1). In general, lipids show lower reproducibility compared to polar metabolites, especially using BuMe.

**Fig. 2:**
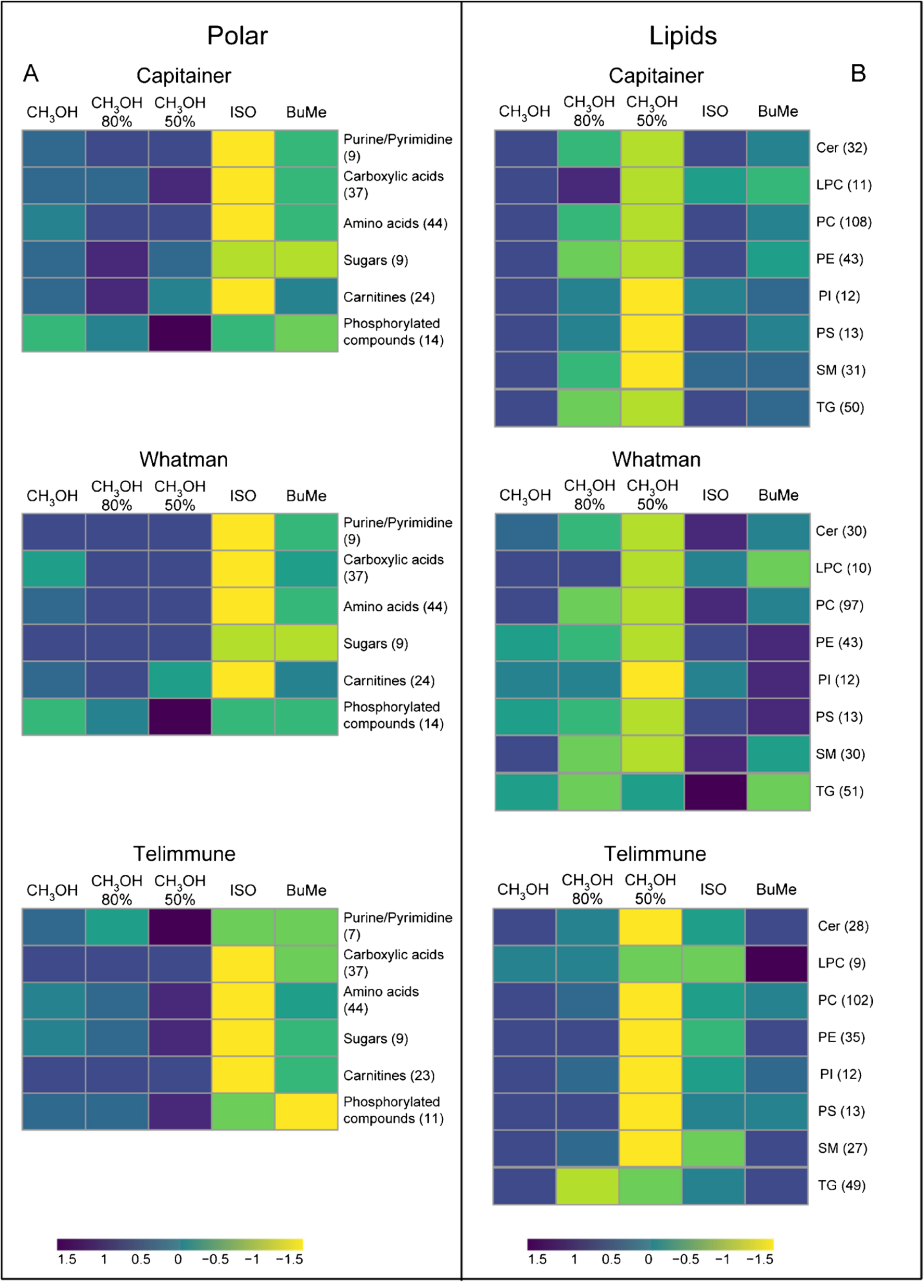
Heatmap extraction. **A** Heatmaps for the extraction of polar metabolite classes for the three devices. **B** Heatmaps for the extraction of lipid classes for the three devices. Each row of the heatmap indicates polar metabolites (**A**) and lipids classes (**B**), and each column the extraction solution. For each class, the color of each cell reflects the average score of each class considering the extraction solution. Shades from violet to yellow represent higher or lower scores, respectively

Comprehensively, for the extraction experiment, 162 polar metabolites and 332 lipids were annotated. The identified polar metabolites were grouped into six classes, purine/pyrimidines, carboxylic acids, amino acids, sugars, carnitines, and phosphorylated compounds, while the identified lipids were divided into eight classes, ceramides (Cer), lysophosphatidylcholines (LPC), phosphatidylcholines (PC), phosphatidylethanolamines (PE), phosphatidylinositols (PI), phosphatidylserines (PS), sphingomyelins (SM), and triacylglycerols (TG). As expected, by evaluating the scores, differences in extraction efficiency were observed among the methods. Extraction methods using methanol-based solvents, CH_3_OH 100%, MeOH 80%, and MeOH 50%, showed higher extraction yields for purine/pyrimidines, carboxylic acids, amino acids, and sugars, while ISO and BuMe exhibited lower performances, and ISO resulted the worst. An interesting pattern was observed for the highly polar metabolites such as phosphorylated compounds: the extraction yield increases proportionally with the percentage of water used in the extraction solution. The observed trends for polar metabolites were similar for Capitainer, Whatman, and Telimmune, as shown in the heatmaps in Fig. 2A. In Fig. 2B, an overview of the lipidome trends of the three devices is shown. As expected, the extraction yield of all lipid classes decreased with the increase of the percentage of water, thus the polarity of the solution. CH_3_OH 100% showed significant extraction yields for all the lipid classes in both DBS and DPS, indicating the efficiency of this solvent for lipid extraction. BuMe proved to be a good alternative with extraction yields comparable to CH_3_OH 100% for Capitainer and Telimmune, while in Whatman, it showed great yields for PE, PI, and PS but not for LPC and TG. Surprisingly, extraction with ISO was not effective for DPS, but was able to extract lipids from whole blood and exhibited the highest extraction yields for all lipid classes in Whatman. CH_3_OH 80% was effective to extract all lipid classes in Telimmune except TG, since their high hydrophobic grade, but for DBS it did not show high yields. The only exception was LPC, a class of lipids with higher polarity: CH_3_OH 80% showed a similar extraction yield compared to CH_3_OH 100% in Whatman, while it performed even better in Capitainer. As expected, we observed notable differences in the extracted polar metabolome and lipidome between DBS and DPS. This could be related to the different biological matrix, different composition of plasma and whole blood, as well as to intrinsic characteristics of the sample, emphasizing the importance of selecting appropriate extraction methods tailored to the sample nature.

**Fig. 3:**
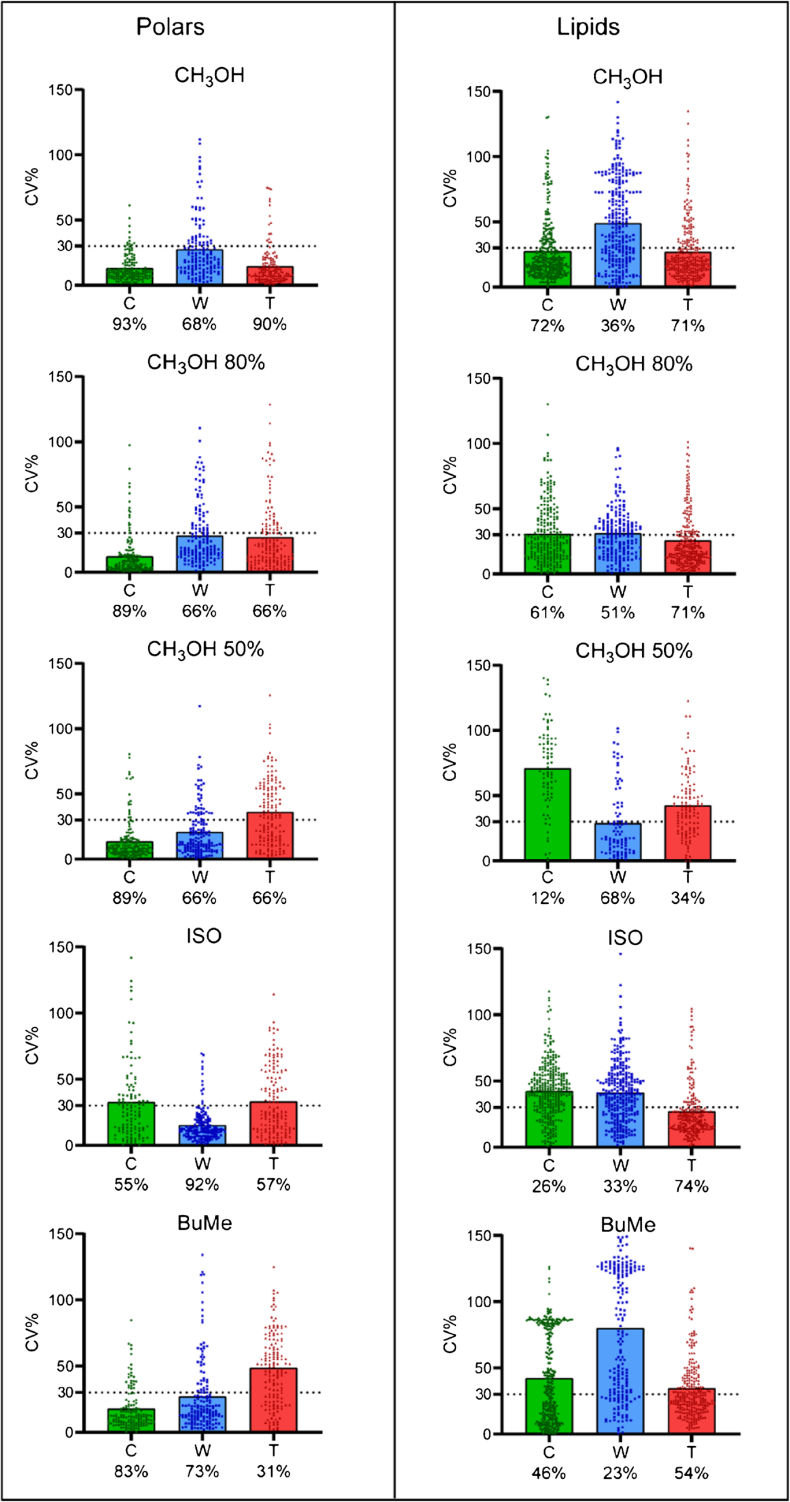
Scatter dot plots to evaluate the reproducibility of each extraction method for polar metabolites and lipids, expressed as CV% (C, Capitainer; W, Whatman; T, Telimmune). Each point represents a single feature

Scatter dot plots were generated to evaluate the reproducibility of each extraction method (Fig. 3). By identifying features with CV% greater than 30%, it is possible to investigate the reproducibility of the extraction solutions within the datasets [18, 19]. For polar metabolites, CH_3_OH 100% exhibits high reproducibility especially for Capitainer and Telimmune, where 93% and 90% of the identified metabolites were characterized by a CV% < 30%. For Whatman, it showed a lower reproducibility with 68% of metabolites with a CV% < 30%. CH_3_OH 80% and CH_3_OH 50% had high reproducibility only in Capitainer (89% of features with CV% < 30%), while it drops to 66% for both Whatman and Telimmune. In contrast, ISO showed high reproducibility only for Whatman (92%), while BuMe for Capitainer was 83%. Looking at lipids, MeOH 100% showed the highest reproducibility for Capitainer and Telimmune, 72% and 71%, respectively. CH_3_OH 80% displayed the same reproducibility as CH_3_OH 100% for Telimmune, while it was lower for Capitainer (61%). CH_3_OH 50% had the best reproducibility in Whatman (68%), while in Capitainer and Telimmune dropped considerably (12% and 34%, respectively). ISO and BuMe had better reproducibility for Telimmune, with 74% and 54% of metabolites with a CV% < 30%, respectively. As expected, extraction with pure organic solvents displayed higher reproducibility for Telimmune. As a general overview, extractions of polar metabolites were more reproducible compared to lipids extractions, and the lowest reproducibility performance was often recorded for Whatman, the only non-volumetric device within the ones tested. Considering the total scores for each extraction method and device (Table 1), in Capitainer, CH_3_OH 50% resulted the best solution for polar metabolite extraction (score 556 ± 5.5) while CH_3_OH 100% recorded the highest score for lipids extraction (993 ± 49.2). In Whatman, CH_3_OH 50% had the highest score (501 ± 61.7) for polar metabolite extraction, while ISO showed the greatest score for the extraction of lipids (808 ± 102), but also a high standard deviation and lower reproducibility. The highest scores found in Telimmune belonged to CH_3_OH 80% (497 ± 55.6) and CH_3_OH (916 ± 86.1) for polar metabolites and lipids, respectively.

**Table 1:** Results for each extraction method are shown for both polar metabolites and lipids in terms of total scores ± standard deviation, CV% intervals, and protein concentration (μg/mL). The total score was calculated as the sum of the scores of each feature for each sample. The total score was then expressed as the average of the scores of the three replicates for each sample. *n.a.*, not available: Protein content was not investigated in Telimmune since the device incorporates a filtration layer that directly separates plasma, and proteins should be retained on top of the membrane

Capitainer B						
		CH_3_OH	CH_3_OH 80%	CH_3_OH 50%	ISO	BuMe
Polar metabolites (%)	Score	459 ± 20.5	531 ± 11.7	556 ± 5.5	191 ± 6.1	343 ± 10.1
	CV% < 10%	48	62	57	21	41
	10% < CV% < 30%	45	27	33	34	41
	CV% > 30%	7	11	10	45	18
Lipids (%)	Score	993 ± 49.2	464 ± 42.7	102 ± 42.3	841 ± 200	692 ± 339
	CV% < 10%	17	14	5	5	22
	10% < CV% < 30%	56	47	6	21	25
	CV% > 30%	27	39	89	74	53
Protein concentration (μg/mL)		110 ± 16.7	137 ± 51.3	1441 ± 75.9	141 ± 35.6	97.6 ± 4.76
CV%		15	37	5	25	5
Whatman 903						
		CH_3_OH	CH_3_OH 80%	CH_3_OH 50%	ISO	BuMe
Polar metabolites (%)	Score	441 ± 32.4	497 ± 15	501 ± 61.7	197 ± 5	359 ± 5.7
	CV% < 10%	19	16	34	36	24
	10% < CV% < 30%	49	50	43	56	48
	CV% > 30%	32	34	23	8	28
Lipids (%)	Score	663 ± 406	326 ± 11.1	145 ± 19.5	808 ± 102	570 ± 369
	CV% < 10%	13	9	28	7	4
	10% < CV% < 30%	23	42	40	27	19
	CV% > 30%	64	49	32	66	77
Protein concentration (μg/mL)		130 ± 8.77	130.2 ± 8.03	620 ± 297	142 ± 10.4	113 ± 6.61
CV%		7	6	48	7	6
Telimmune DUO						
		CH_3_OH	CH_3_OH 80%	CH_3_OH 50%	ISO	BuMe
Polar metabolites (%)	Score	393 ± 5.7	497 ± 55.6	484 ± 29.8	193 ± 11.8	271 ± 6.7
	CV% < 10%	51	29	13	21	9
	10% < CV% < 30%	39	37	32	36	22
	CV% > 30%	10	34	55	43	69
Lipids (%)	Score	916 ± 86.1	702 ± 34	166 ± 46	499 ± 73.6	782 ± 136
	CV% < 10%	17	20	6	9	6
	10% < CV% < 30%	54	51	28	65	48
	CV% > 30%	29	29	66	26	46
Protein concentration (μg/mL)		n.a	n.a	n.a	n.a	n.a
CV%		n.a	n.a	n.a	n.a	n.a

**Fig. 4:**
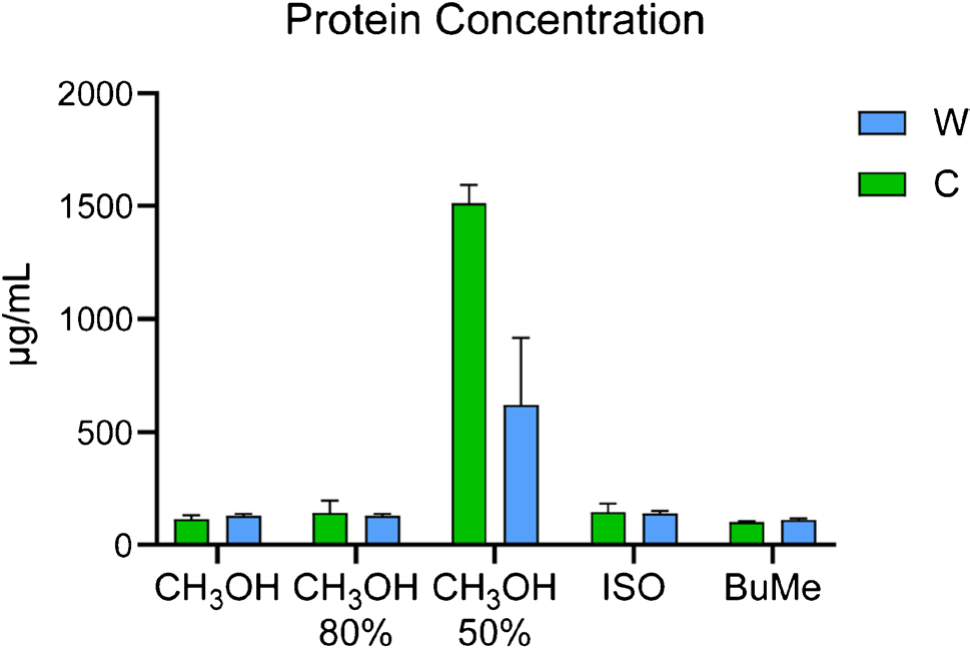
BCA quantification. Protein concentrations, expressed in μg/mL, quantified with BCA assay for each extraction method in DBS samples (C, Capitainer; W, Whatman). Protein concentration in DBS using CH_3_OH 50% resulted statistically different compared to the other four extraction solution (*p*-value < 0.0001)

Quantitative BCA protein assay was performed on DBS samples to evaluate the protein concentrations in samples, in particular hemoglobin contained in red blood cells, extracted with the five extraction solutions. According to the results shown in Fig. 4, elevated protein concentration was reported in samples extracted with CH_3_OH 50%, 1500 μg/mL in Capitainer, and 600 μg/mL in Whatman, while the other extraction solutions showed a comparable protein concentration, with a range of 100–150 μg/mL. These data suggested that using CH_3_OH 50% with both Capitainer and Whatman devices, a further filtration step is mandatory to remove hemoglobin before metabolomics/lipidomics analysis. However, a smaller amount of proteins was always detected in all samples analyzed. Therefore, to minimize ion suppression and/or interferences due to residual amount of protein in the sample, we always suggest filtration for the analysis of DBS.

BCA assay was not performed on DPS due to the intrinsic features of the Telimmune device. Red blood cells are already filtered out without centrifugation thanks to the filtration layer that enables direct separation of plasma from whole blood drops using a combination of filtration and adsorption.

CH_3_OH-based solutions consistently showed higher extraction efficiencies for polar metabolite classes, which increased proportionally with the percentage of water in the solution. These results are consistent with previous studies in literature [12, 13, 20], highlighting the good performance of these solvents in the extraction of polar metabolites and less hydrophobic lipids. CH_3_OH 50% showed the highest extraction yields for polar metabolites for all three devices, but it was also the solution that extracted the highest protein concentration according to quantitative BCA assay, which is of course a limitation for metabolomics and lipidomics analysis. For lipids, the best performances with Capitainer and Telimmune were obtained with CH_3_OH 100%. Conversely, for Whatman device, the best results were obtained with samples extracted with ISO, with CH_3_OH being a viable alternative. For lipidomics analysis, however, extraction methods using pure organic solvents, CH_3_OH, ISO, and BuMe, showed higher extraction performance. This result is also consistent with the literature: as described in other studies, the most commonly chosen extraction solvents for lipidomics analysis are usually pure organic solvents [21, 22]. Considering the reproducibility of the extraction method, measured as CV%, MeOH 100% lead to the highest reproducibility for polar metabolites in Capitainer and Telimmune. For Whatman, this reproducibility performance was achieved for samples extracted with ISO. Regarding the variability of extraction methods for lipids, Capitainer samples extracted with CH_3_OH showed the highest reproducibility, while CH_3_OH 50% extraction allowed for better reproducibility in Whatman. For Telimmune, ISO proved to be the solvent with the best reproducibility. Overall, it is essential to select the most suitable blood microsampling device to maximize analyte coverage, hence ensuring comprehensive evaluation and meaningful insights into the metabolic profile. Our results showed significant differences in the number of metabolites and lipids detected with different DBS/DPS devices. By observing the performance of solvent-based extraction methods on different sample matrices, the need for tailored extraction strategies to optimize lipidomics or metabolomics analysis based on sample type should be highlighted. These results suggest that when working with DBS/DPS, it is most convenient to perform both metabolomics and lipidomics on the same spot, by extraction with CH_3_OH. Of course, this is a compromise. When extracting with pure CH_3_OH, several polar metabolites, in particular phosphorylated compounds, are not properly recovered. Phosphorylated compounds mainly represent the intracellular polar metabolome extracted from the red blood cells.

**Fig. 5:**
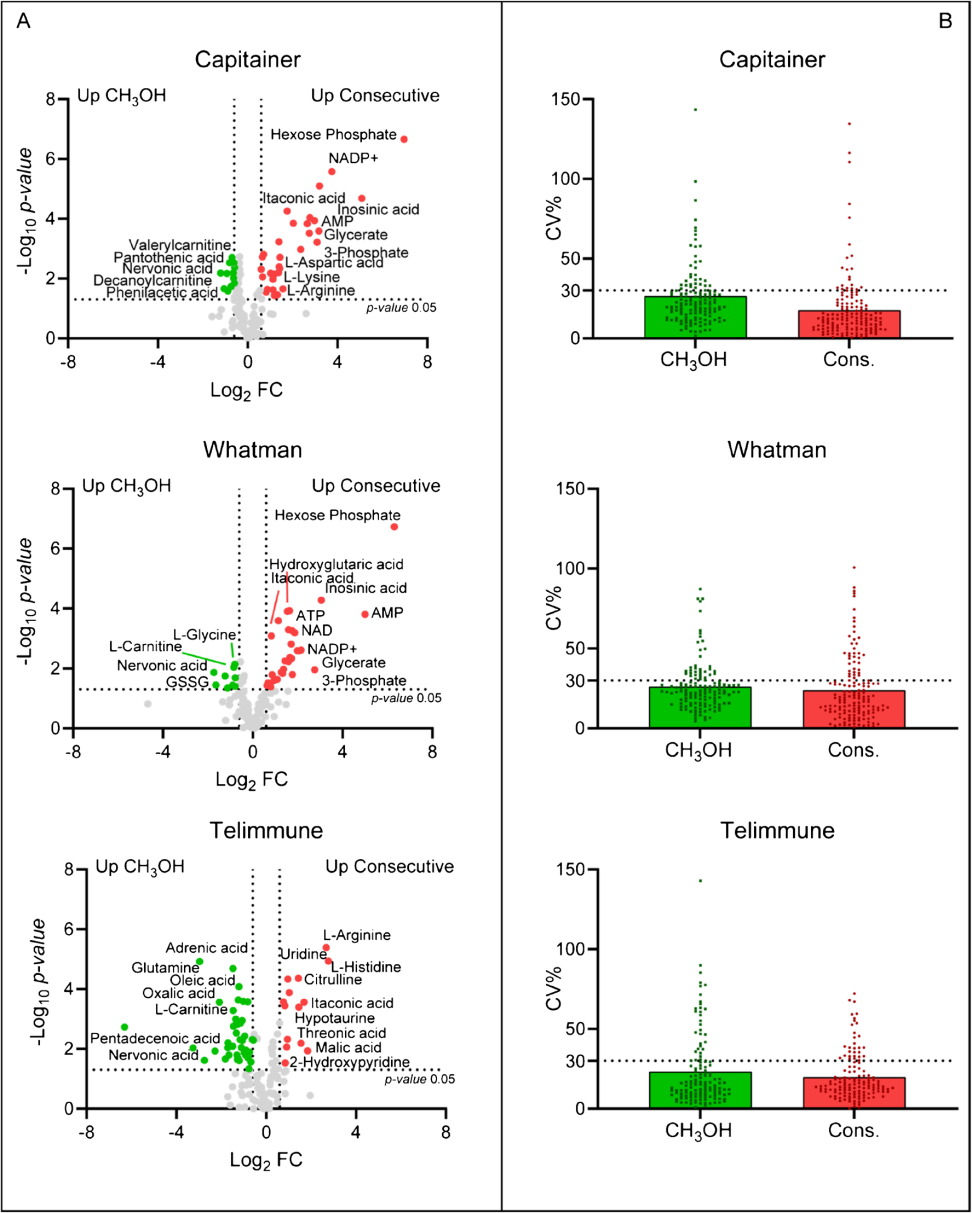
Consecutive extraction. **A** Volcano plots (*p*-value threshold 0.05, FC 1.5). **B** Scatter dot plots. Evaluation of the reproducibility of CH_3_OH extraction compared to consecutive extraction, in terms of CV%

However, an additional coverage of the red blood cells polar metabolome might be useful information that can be exploited in several fields when working with DBS, and it is to preserve it. For this reason, we decided to perform a two-step consecutive extraction to improve the polar metabolome coverage. The first step was performed with CH_3_OH extracting both polar and lipid metabolites, followed by a second extraction step with H_2_O to improve polar metabolites coverage. According to the results, the consecutive extraction increased the coverage of the polar metabolome and in particular for phosphorylated compounds in DBS, as can be seen in the volcano plots in Fig. 5. In addition, the number of identified polar metabolites with a CV% < 30% increased in both Capitainer and Whatman, indicating better reproducibility compared to CH_3_OH extraction. No significant difference was observed in Telimmune, suggesting that consecutive extraction is probably not required for DPS (Fig. 5), since it does not contain intracellular polar intracellular metabolites.

### Evaluation of short-term stability

DBS are used to collect capillary blood, but there can be a variable time frame (from a few hours to several days) between collection and extraction of the metabolome, especially true when sampling is done remotely. For this reason, it is important to evaluate the short-term stability of DBS/DPS and to understand how storage time affects the metabolome stability and coverage. To address this question, we designed an experiment in which 18 samples (six for each device tested) were spotted and left at RT for 1 to 5 days, respectively, before storage at − 80 °C, while samples at day 0 were immediately stored at − 80 °C.

**Fig. 6:**
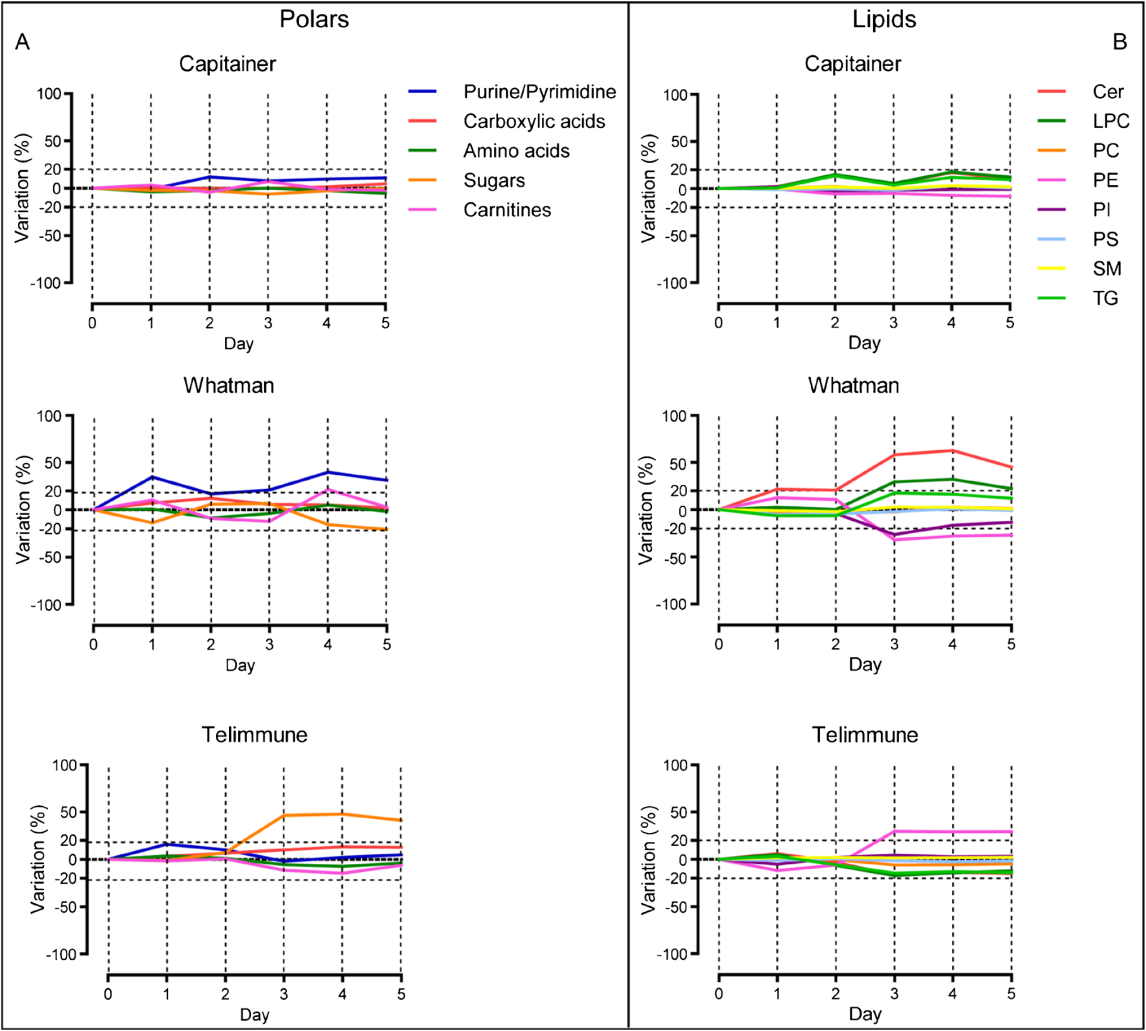
Variation to baseline. The plots show the variation % of polar metabolite classes (**A**) and lipid classes (**B**) within the 5 days. Deviations within ± 20% are considered acceptable; thus, classes with a variation in this range are considered stable

Plots in Fig. 6 show the relationship between the variation to baseline and the number of days at RT. With an acceptable deviation of ± 20%, both polar metabolites and lipids can be considered stable for up to 5 days at RT in Capitainer. In Whatman, all polar metabolite classes are stable up to 5 days, with the exception of purine/pyrimidine, which were stable at RT only at day 0. Lipids were less stable overall in Whatman: Cer, LPC, PI, and PE were stable up to day 2 at RT. Ceramides in particular reached a variation of more than 50% after day 3. In Telimmune, both polar metabolite and lipid classes proved to be stable up to 5 days. The only exception for polar metabolites were sugars, which were stable up to day 2 before reaching a variation of around 50%. For lipids, PE were stable up to day 2 at RT.

The stability appears to depend on both the chemical class and metabolite. Several solutions can be employed to stabilize blood samples. Lowering the temperature is the most common method used for stabilizing small molecules [23]. Moreover, pH adjustment, the addition of inhibitors and/or antioxidants, and the use of sealed bags with desiccants can be implied to improve the stability [24]. However, one of the main challenges in metabolomics and lipidomics studies is to find a solution to stabilize the whole metabolome and lipidome. Currently, lowering the storage temperature is the most used and the most successful approach for this purpose. Overall, improving the short-term stability of DBS/DPS samples is critical to ensure minimal degradation at RT. Limited stability is likely linked to a combination of different factors, including oxidation, enzymatic degradation, and degradation at ambient humidity and temperature conditions [23]. Therefore, further studies are necessary to investigate different storage conditions or approaches to ensure short-term stability of DBS and DPS.

## Conclusion

In this study, we evaluated different extraction methods for DBS and DPS and optimized the extraction protocol to perform both metabolomics and lipidomics analyses on the same spot. Overall, the results showed that the best compromise was the extraction with pure methanol for both lipids and polar metabolites. However, in order to improve the coverage of the extracted polar metabolome, a two-step protocol can be used that combines extraction with methanol (providing the lipidome) followed by a further consecutive extraction with water (providing polar metabolome) on the same spot. The two-step consecutive extraction was shown to improve the reproducibility in Capitainer and Whatman, while it did not show any significant change in Telimmune, meaning that probably for DPS the consecutive extraction is not needed. Moreover, protein filtration is suggested for both Capitainer and Whatman to avoid further protein interference.

The short-term stability of DBS and DPS at RT was evaluated for 5 days. In Capitainer, both polar metabolites and lipids were stable up to 5 days at RT. For Whatman and Telimmune, some significant changes for some metabolite/lipid classes were observed after 3 days. These results suggest the need for cold-chain storage within 3 days from the sampling, in order to avoid sample degradation when working with Whatman and Telimmune devices. If necessary, it could be also useful to assess the stability of selected metabolites or classes of selected metabolites. It is important to note that all the considerations resulted from this study are related to untargeted metabolomics and lipidomics. When performing a targeted analysis, it is necessary to evaluate specific classes of compounds that might be interesting.

According to our results, Capitainer showed the best performance in terms of extraction, reproducibility, and short-term stability. This might be related to the fact that it is a quantitative device which was implemented to solve some of the challenges related to non-quantitative devices, such as Whatman. One of these issues can be due to the HCT bias which affects the inter- and intra-sample variability. On the other hand, Whatman has been widely used to collect blood with different applications, since its first use in the 1960s and it is still the most affordable option. Telimmune is currently the only available microsampling device that allows direct separation of plasma from capillary blood. Comprehensively, it is important to choose the microsampling device considering all the variables, including the costs and the type of application. Indeed, these advancements create new opportunities for utilizing lipidomics and metabolomics in diagnostics, biomarker discovery, and personalized medicine. Furthermore, these improvements emphasize the importance of incorporating robust methodologies into clinical workflows to support precision medicine and broaden the use of dried blood plasma spot analyses. For future studies, it could be interesting to perform a comparison by performing untargeted metabolomics and lipidomics analysis on DPS and on plasma samples.
